# The current landscape of microRNAs (miRNAs) in bacterial pneumonia: opportunities and challenges

**DOI:** 10.1186/s11658-022-00368-y

**Published:** 2022-08-19

**Authors:** Fan Zhang, Yunxin Zhou, Junying Ding

**Affiliations:** Beijing Key Laboratory of Basic Research With Traditional Chinese Medicine On Infectious Diseases, Beijing Institute of Chinese Medicine, Beijing Hospital of Traditional Chinese Medicine, Capital Medical University, Beijing, 100010 China

**Keywords:** miRNA, Bacterial pneumonia, Host–pathogen, Sensitivity, Specificity, Off-target effect

## Abstract

MicroRNAs (miRNAs), which were initially discovered in *Caenorhabditis elegans*, can regulate gene expression by recognizing cognate sequences and interfering with the transcriptional or translational machinery. The application of bioinformatics tools for structural analysis and target prediction has largely driven the investigation of certain miRNAs. Notably, it has been found that certain miRNAs which are widely involved in the inflammatory response and immune regulation are closely associated with the occurrence, development, and outcome of bacterial pneumonia. It has been shown that certain miRNA techniques can be used to identify related targets and explore associated signal transduction pathways. This enhances the understanding of bacterial pneumonia, notably for “refractory” or drug-resistant bacterial pneumonia. Although these miRNA-based methods may provide a basis for the clinical diagnosis and treatment of this disease, they still face various challenges, such as low sensitivity, poor specificity, low silencing efficiency, off-target effects, and toxic reactions. The opportunities and challenges of these methods have been completely reviewed, notably in bacterial pneumonia. With the continuous improvement of the current technology, the miRNA-based methods may surmount the aforementioned limitations, providing promising support for the clinical diagnosis and treatment of “refractory” or drug-resistant bacterial pneumonia.

## Introduction

Infectious pneumonia is an inflammation of the terminal airway, alveoli, or interstitium caused by pathogenic microorganisms [1, 2]. Pneumonia caused by bacterial infection is the most common form of pneumonia [3]. In the past two decades, the mortality rate of bacterial pneumonia has increased due to the decline in air quality, the increase in the number of harmful factors from the environment [4], and the extensive use of antibiotics [5]. The development of novel antibiotics occurs at a slower rate than that of the evolution of bacterial resistance. As a result, the mortality rate from pneumonia has been increasing in recent years [6], creating an urgent need to clarify the pathogenic mechanisms, improve the diagnostic measures, and enhance the pertinent treatment strategies for bacterial pneumonia.

RNA interference (RNAi) is a process in which double-stranded RNA (dsRNA) induces specific gene silencing and inhibits the expression of a target gene [7]. RNAi includes endogenous miRNAs and exogenous small interfering RNAs (siRNAs) [8]. miRNAs can induce the inhibition of target messenger RNA (mRNA) expression at the protein level or the degradation of the target mRNA at the transcriptional or translational level. They can also negatively regulate multiple signaling pathways [9]. The function and the roles of the target genes in the occurrence, development, treatment, and prognosis of diseases have been clarified by comparing the gene phenotypes with or without the miRNAs [10, 11].

The application of miRNAs is considered more sophisticated than traditional diagnostic or therapeutic methods [12]. A previous study has shown that well-designed miRNAs which are used as therapeutic targets can provide long-term silencing in experimental studies while reducing toxicity and side effects [13]. However, the miRNA-based methods and experimental techniques face several challenges, such as low sensitivity, poor specificity, low silencing efficiency, off-target effects, and toxic reactions [14]. In the present study, the application of specific miRNA-based methods in the pathogenesis, diagnosis, and prognosis of bacterial infections was reviewed. These methods are crucial for studying the mechanism of bacterial pneumonia and identifying diagnostic and therapeutic targets that could prevent "refractory" pneumonia caused by bacterial pneumonia. Despite the limitations and challenges of the miRNA-based methods, they are still considered a promising strategy to identify the targets for the diagnosis and/or treatment of bacterial pneumonia.

### Biosynthesis, functional modes, and therapeutic approaches

miRNAs are a group of endogenous non-protein-coding small RNA molecules with a length of approximately 20–22 nucleotides (nt) [15]. The function of miRNAs involves the negative regulation of gene expression at the post-transcriptional level [16]. Specifically, miRNAs are combined with the 3′-untranslated region (3′-UTR) of a target mRNA, which causes inhibition of translation (incomplete complementary pairing) or target mRNA degradation (complete complementary pairing) [17, 18]. This negative regulation can inhibit or promote the expression levels of related genes via the activation of particular signaling pathways which may affect the occurrence and development of certain diseases [19].

**Fig. 1:**
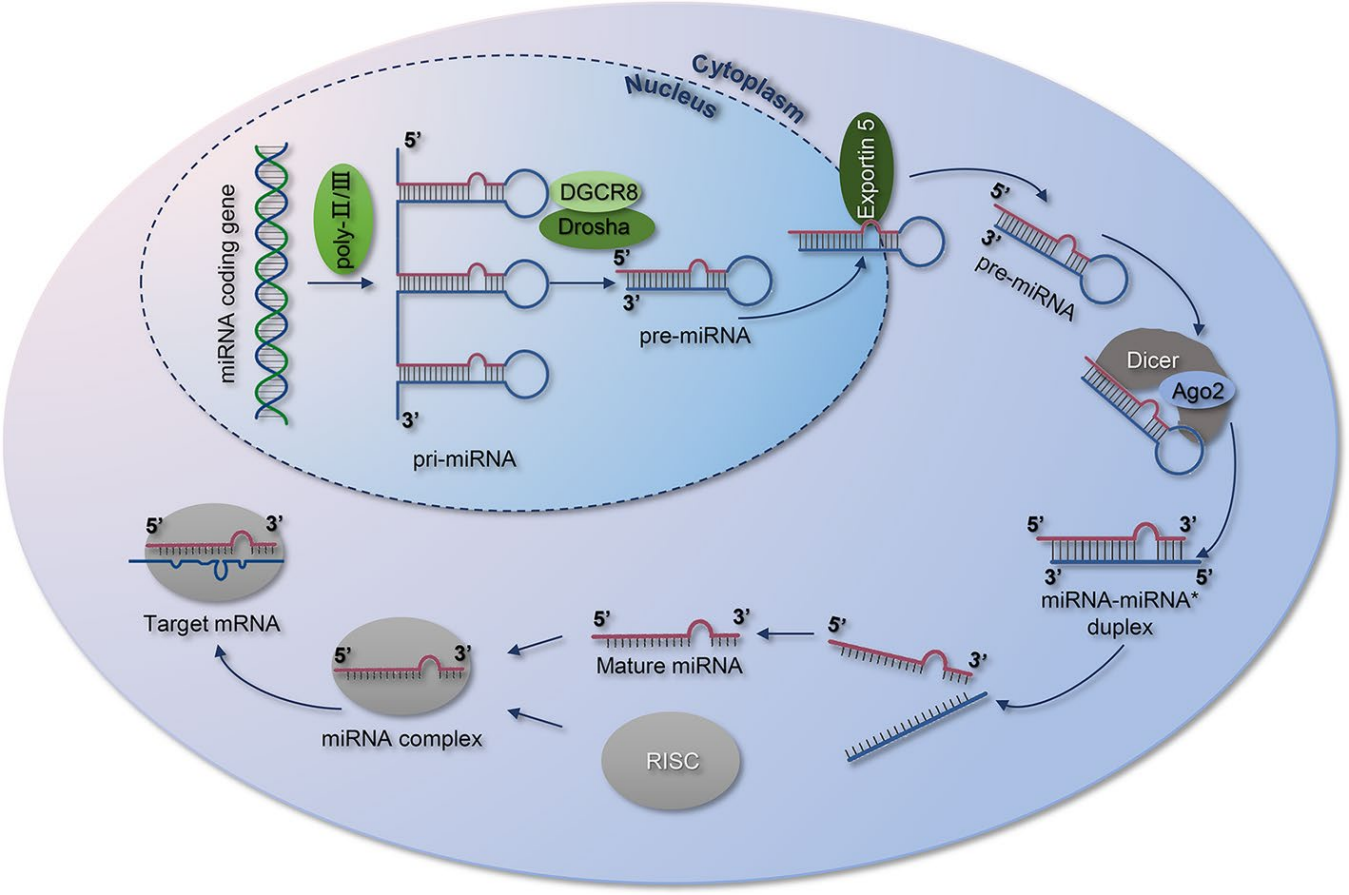
The biosynthesis process of microRNAs. This multistep maturation process includes the production of the pri-miRNA transcript by RNA Pol II&III and cleavage of the pri-miRNA by the microprocessor complex (Drosha and DGCR8) in the nucleus. The resulting pre-miRNAs are exported from the nucleus via Exportin-5. In the cytoplasm, the RNase Dicer forms a complex and cleaves the pre-miRNA hairpin to its mature length. The functional strand of the mature miRNA is combined with Ago2 into the RISC, where it guides it to induce silencing of target mRNAs through mRNA cleavage, translational repression, or deadenylation

The biosynthesis of miRNAs is a multistep process, which comprises three main stages (Fig. 1). Initially, the miRNA coding genes are transcribed as primary miRNAs (pri-miRNAs) by RNA polymerase II and III (Pol II&III) in the nucleus. The pri-miRNAs are subsequently cleaved by the microprocessor complex (Drosha and DGCR8) into a stem-loop structure called precursor miRNAs (pre-miRNAs) [20]. Following this step, pre-miRNAs are transported into the cytoplasm via Exportin-5 from the nucleus [21]. In the cytoplasm, the pre-miRNA hairpin is cut into double-stranded mature miRNA by the RNase Dicer. Subsequently, mature miRNAs are combined with Ago2 to form the active RNA-induced silencing complex (RISC) [22]. Finally, the active complex interacts with the 3'-UTR of the target mRNA through complementary base pairing, which inhibits the translation or promotes the degradation of the target mRNAs, thus exerting the post-transcriptional regulation of gene expression [18, 23].

**Fig. 2:**
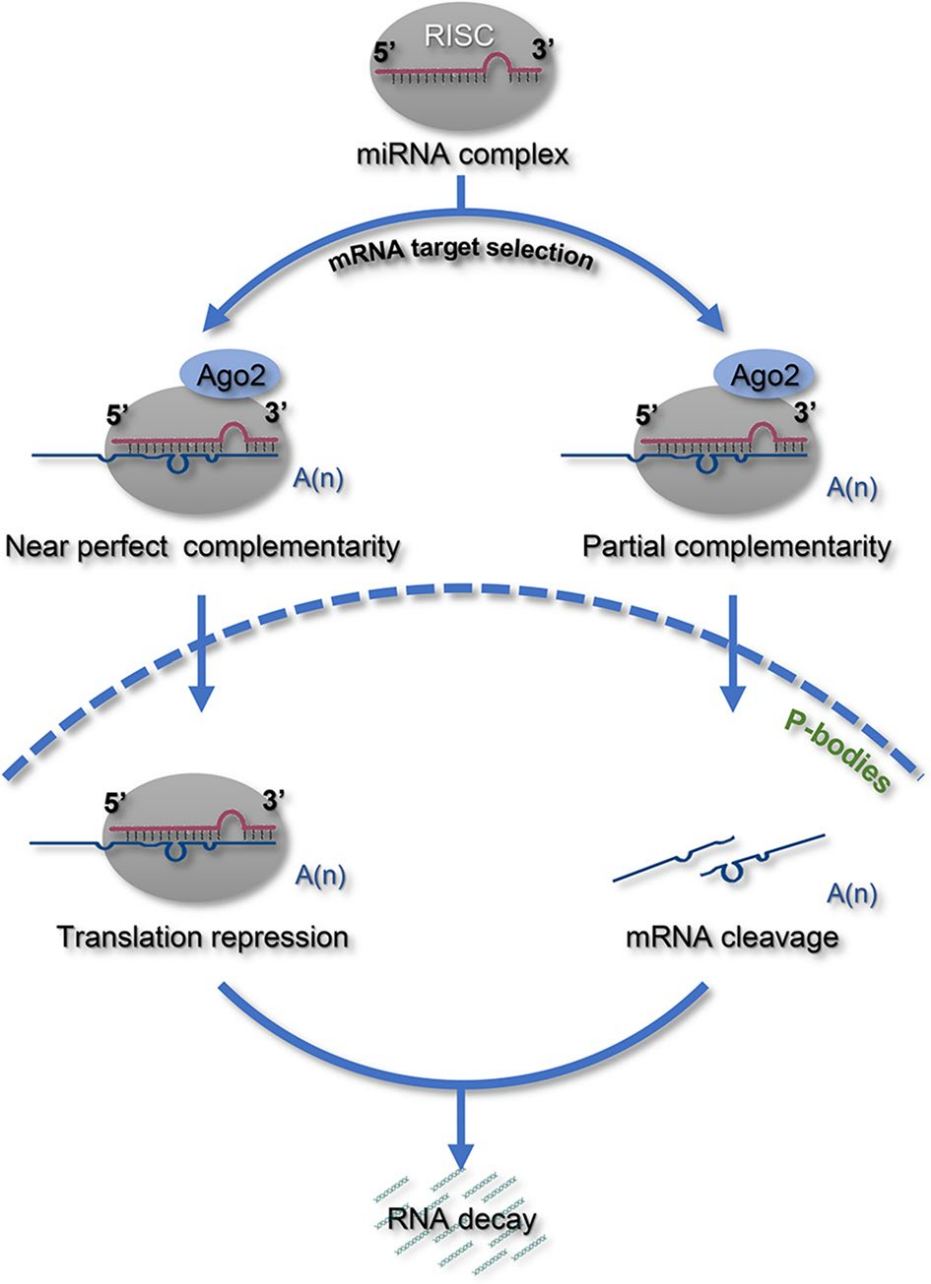
Schematic diagram of miRNA-mediated translational repression. In RISC, miRNA-mediated downregulation of target gene activity occurs via two modes: target mRNA cleavage or translational inhibition. The choice is made based on the degree of complementarity between the miRNA and target gene in combination with an Argonaute family protein. Near-perfect complementarity results in cleavage, followed by general RNA degradation of the targets, whereas partial complementarity causes translational inhibition. miRNA-targeted mRNAs can be sequestered on polysomes or recruited to P-bodies where they are depleted of the translation machinery and eventually degraded

The function of miRNAs involves the two following main modes: miRNA degradation or translational inhibition [24] (Fig. 2). The difference between the two modes is the degree of complementarity between miRNAs and their target mRNAs [25]. Generally, if the miRNAs are perfectly aligned to the target mRNAs, they trigger the degradation of the target mRNA. When the alignment includes nonspecific parts, the miRNA is only able to inhibit the translation of the target mRNA [26].

As a research tool, the miRNA-based methods include the two following major approaches: Inhibition therapy or replacement therapy [27]. The former uses miRNA inhibitors to downregulate the aberrant overexpression of miRNAs [28]. The miRNA inhibitors could effectively antagonize the inhibition of protein The miRNA inhibitors can effectively antagonize the inhibition of protein expression from the upregulation of miRNAs. To date, complementary antisense oligonucleotides and miRNA sponges have been used to inhibit miRNA expression by interfering with the miRNA-induced silencing complex (miRISC) [29]. Similarly, the second approach utilizes synthetic miRNA mimics to restore the downregulated miRNA activity [30]. In order to achieve similar biological functions to the miRNAs in vivo, miRNA mimics should be combined with the RISC complex. In this way, the miRNA mimics can affect the ability of miRNAs to target specific mRNAs [31].

### Function and mechanism on host and/or pathogen

**Fig. 3:**
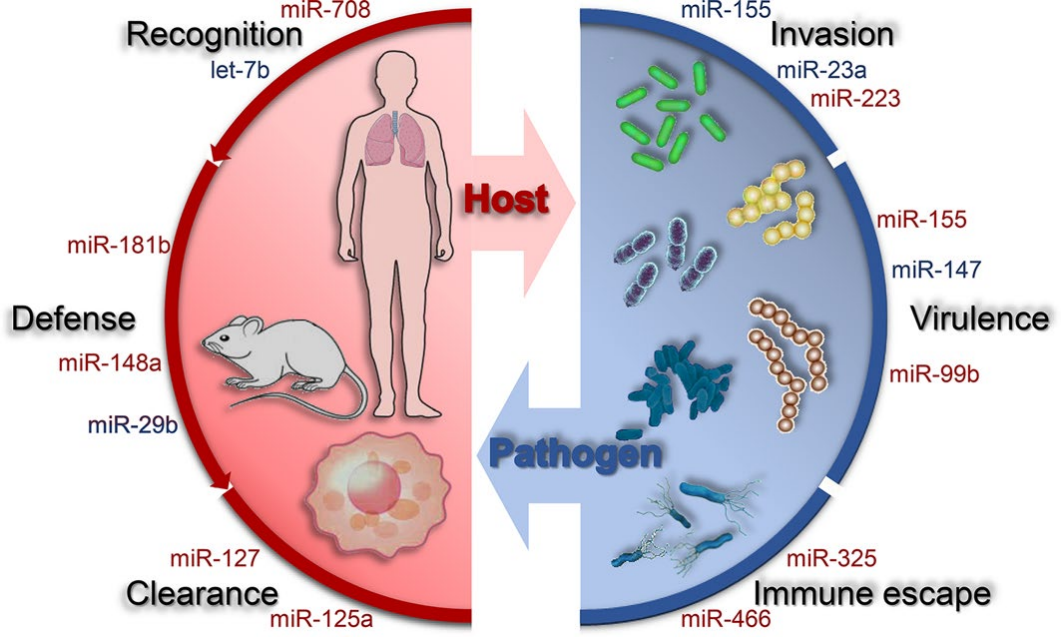
The interactions between the host and the pathogen. As a part of host innate immunity and adaptive immune response, pathogen infection is neutralized by miRNAs by regulating the recognition of the pathogen, the defense against it, and its clearance. At the same time, miRNAs can inhibit the host immune response and induce host infection by enhancing the invasion of pathogens, moderating the virulence of pathogens, and aiding their immune escape

In recent years, bacterial resistance caused by the abuse of antibiotics has been increasing [32]. Moreover, the identification of novel antibiotics is facilitated by backward R&D technology [33]. Under these circumstances, the most lethal and debilitating bacterial infection remains the greatest worldwide challenge to public health [5]. Therefore, alternative antibacterial strategies are urgently required. The roles of miRNAs in bacterial infection have been extensively studied in the past few years [34, 35]. With regard to post-transcriptional gene regulation, miRNAs have been shown to play a pivotal role in the complex interaction between host cells and bacterial pathogens [36, 37] (Fig. 3).

### Assisting the host in resisting pathogen infection

Pathogen infection is neutralized by miRNAs, which are considered a part of the host’s innate and adaptive immunity [38, 39]. Specifically, the recognition of the pathogen, the defense against it, and its clearance have been shown to be involved in the process of miRNA-neutralizing pathogen infection (Table 1).

**Table 1:** miRNAs involved in assisting the host in resisting pathogen infection

	Shuttle	Expression	Target	Species/Cells	Functions	References
Recognition	miR-708-5p	↑	TLR4	Human/HTP-1, U937	Enhancing the vitality of intracellular bacterial Regulating the secretion of inflammatory factors	[44]
	let-7b	↓	TLR4	Mice/T84	Promoting the release of proinflammatory cytokines Inducing excessive mucosal immune response	[45]
Defense	miR-181b	↑	LXA_4_	Human/16HBE14o-, MΦs	Dampening neutrophil infiltration Reducing the release of proinflammatory mediators Limiting collateral tissue damage	[50]
	miR-148a-3p	↑	PTEN	Mice/BM	Promoting M1 macrophage activation and inflammatory factor secretion through NF-κB Enhancing the ability of macrophages to engulf and kill bacteria via ROS production	[52]
	miR-29b-2-5p	↑	UNC5C	Human/HeLa-229	Promoting the filopodia formation Increasing the bacterial capture Enhancing the bacterial invasion	[39]
	miR-29b-2-5p	↓	UNC5C	Human/HeLa-229	Favor the balanced intracellular replication to avoid premature cell death Conducing dissemination to neighboring cells	[39]
Clearance	miR-127	↑	A20	Mice/RAW264.7, AMs, BMDMs human/THP-1	Upregulating the proinflammatory cytokines and anti-bacterial molecules in macrophages Enhancing the clearance of bacteria Promoting the phosphorylation and ubiquitination of STAT3	[55]
	miR-125a-5p	↑	STAT3	Human/THP-1	Decreasing the autophagy of macrophages Improving the intracellular bactericidal survival	[56]

“↑” increased; “↓” decreased

### Regulating the host’s recognition of the pathogen

A class of microbial components termed pathogen-associated molecular patterns (PAMPs) has been recognized by the host through pattern-recognition receptors (PRRs) [40]. This is the first step to initiate host innate immunity [41]. Membrane-bound toll-like receptors (TLRs) are a subset of PRRs [42]. Subsequently, proinflammatory signaling cascades can be activated by TLRs following host recognition [43]. Consequently, TLRs, which are the receptors of PRRs, play a vital role in mediating host innate immunity and activating proinflammatory signaling cascades by recognizing microbial components termed PAMPs. Recent studies have shown that the ability of the host to recognize pathogens can be regulated by miRNAs through TLRs.

The host immune response is activated by miR-708-5p through recognition of its target toll-like receptor 4 (TLR4) [44]. The application of the miRNA inhibition and replacement therapy has shown that miR-708-5p expression is upregulated in macrophages with *Mycobacterium tuberculosis* (*M. tuberculosis*). Furthermore, the secretion of inflammatory factors of *M. tuberculosis* is negatively regulated by miR-708-5p. Specifically, it was shown that the survival rate of intracellular *M. tuberculosis* was significantly increased following the transfection of the cells with miR-708-5p mimics, while it was substantially decreased following the application of miR-708-5p inhibitors. In addition, the difference in the survival rate of *M. tuberculosis* is consistent with the expression of the downstream inflammatory factors mediated by TLR4. Therefore, miR-708-5p regulates the ability of the host to recognize pathogens by targeting TLR4.

Let-7b controls the release of proinflammatory cytokines by modulating the expression of TLR4 [45]. Specifically, the overexpression of let-7b decreased the secretion of proinflammatory cytokines via modulating TLR4 expression, which improved the severity of colitis. By contrast, let-7b inhibitors exhibited the opposite effects. Collectively, the results indicated that let-7b is involved in regulating the excessive mucosal immune response of adherent-invasive *Escherichia coli* (AiEC) to the intestinal flora. Therefore, it may be a potential therapeutic target for Crohn’s disease (CD).

### Modulating the host’s defense against pathogen invasion

Innate and adaptive immunity are the two main mechanisms of the host immune system used to combat invading pathogens [46]. Generally, innate immunity provides a first-line protection to the host, and adaptive immunity mounts specific attacks against invading pathogens [47]. Therefore, the ability of the host to combat invading pathogens is closely related to the status of the immune system [48]. The immune status of the host against pathogen invasion can be modulated by specific miRNAs [49].

Bacterial invasion is inhibited by miR-181b through modulation of the LXA4/FPR2-LXA4 circuit [50]. The biological activity of LXA4 is inhibited by the high expression of miR-181b in cystic fibrosis (CF) cells. LXA4, the ALX4/FPR2 agonist, can dampen neutrophil infiltration, and reduce the release of proinflammatory mediators, which limit collateral tissue damage. In general, *Pseudomonas aeruginosa* (*P. aeruginosa*) is considered to be a colonizing bacterium in the airways of the majority of CF patients [51]. By transfection of monocyte-derived macrophages with miR-181b inhibitors, the biological activity of LXA4 was enhanced. The colonization of *P. aeruginosa* in CF cells was controlled. In brief, this study demonstrated that the targeting of miR-181b enhanced the anti-inflammatory and anti-microbial defenses.

Bacterial invasion is inhibited by the induction of miR-148a-3p expression through the PTEN/AKT pathway [52]. As a novel downstream molecule of Notch signaling, miR-148a-3p promotes the differentiation of monocytes into macrophages. Moreover, the M1 or M2 polarization of macrophages is promoted or inhibited, respectively, by miR-148a-3p via Notch activation. In addition, the overexpression of miR-148a-3p in macrophages increased the production of reactive oxygen species (ROS) by targeting PTEN. This may be the mechanism of the miRNA-mediated defense against bacterial invasion. Therefore, miR-148a-3p can protect against bacterial invasion via activation of the PTEN/AKT pathway.

miR-29b-2-5p plays a dual regulatory role in the regulation of bacterial infection [39]. The expression levels of miR-29b-2-5p are relatively high at the initiation of the infection. miR-29b-2-5p promoted filopodia formation, increased the bacterial capture, and enhanced bacterial invasion by inhibiting the expression of the target gene UNC5C. With the replication of the bacteria, the expression levels of miR-29b-2-5p were gradually decreased at late time periods post-infection. In order to avoid premature cell death, intracellular replication must be balanced. This also prevents bacterial spread to neighboring cells. Collectively, these characteristics could effectively reduce excessive death and maintain cellular homeostasis.

### Adjusting the host’s clearance of the pathogen

Macrophages are considered to be one of the most momentous innate immune cell classes. They play important roles in clearing infected microbes [53]. The inflammatory response can be initiated by macrophages via the release of proinflammatory cytokines. Therefore, macrophages can phagocytose and kill specific pathogens [54]. The phagocytosis of macrophages can be adjusted by miRNAs to moderate the clearance of the pathogen.

Antibacterial innate immunity is modulated by miR-127 via the regulation of the A20/STAT3 axis [55]. A previous study indicated that the expression levels of miR-127 were induced by *Staphylococcus aureus* (*S. aureus*) infection. The expression levels of certain proinflammatory cytokines and antibacterial molecules present in macrophages were upregulated by miR-127. This enhanced bacterial clearance. In addition, bacterial pneumonia was alleviated with reduced bacterial burden in mice treated with miR-127 mimics. Moreover, the phosphorylation and ubiquitination of STAT3 were upregulated by miR-127 via inhibiting the expression of A20. The effects of miR-127 on the production of specific antibacterial mediators were largely abolished following the overexpression of A20 or treatment with STAT3 inhibitors. Therefore, miR-127 was confirmed to be a specific target in modulating the clearance of pathogens via targeting the A20/STAT3 axis.

The host’s innate immunity has been shown to be regulated by miR-125a-5p via targeting STAT3 and enhancing autophagy [56]. Specifically, miR-125a-5p was highly expressed in macrophages as a response to bacterial infections. Furthermore, the expression levels of STAT3 in THP-1 cells were inhibited by overexpression of miR-125a-5p. By contrast, the miR-125a-5p inhibitors exhibited the opposite effect, whereas inhibition of STAT3 enhanced autophagy in macrophages. Therefore, the induction of autophagy was increased and the bacterial survival was decreased following the overexpression of miR-125a-5p. Conversely, these effects were reversed by co-transfection with miR-125a-5p mimics and the STAT3 expressing construct. Collectively, these findings indicated that miR-125a-5p participates in the modulation of the clearance of pathogens by targeting STAT3 and enhancing autophagy.

### Impeding the pathogen to induce host infection

The host immune response can be suppressed by miRNAs which induce pathogen infection [39]. Notably, the increased invasion of pathogens, the modulation of the pathogen virulence, and the induction of the immune escape of the pathogens are the three main aspects of miRNAs that are involved in the suppression of the host immune response (Table 2).

**Table 2:** miRNAs involved in impeding the pathogen to induce host infection

	Shuttle	Expression	Target	Species/Cells	Functions	References
Invasion	miR-155	↓	HMGN2	Human/A549, HBE16	Promoting the function of integrin α5β1 Increasing the polymerization of actin Regulating the adhesion of bacteria	[59]
	miR-23a	↓	HMGN2	Human/A549, HBE16	Modulating the expression of integrin α5β1 Regulating the adhesion of bacteria	[59]
	miR-223-3p	↑	CagA	Human/AGS	Regulating the colonization of bacteria Increasing the cell proliferation and migration	[60]
	miR-223-3p	↑	NLRP3	Human/THP-1	Mediating the concentration of bacteria Adjusting inflammasome function and responses	[62]
Virulence	miR-155	↑	PepO	Mice/PEMs	Enhancing the phagocytosis of macrophages Promoting host defense response to bacteria	[67]
	miR-147-3p	↓	Esx-1	Mice/RAW264.7	Increasing the survival rate of bacteria	[69]
	miR-155	↑	Rv2346c	Human/U937 Mice/RAW264.7	Increasing the bacterial load and lung injury Enhancing the secretion of virulence factor	[71]
	miR-99b	↑	Rv2346c	Human/U937 Mice/RAW264.7	Increasing the bacterial load and lung injury Enhancing the secretion of virulence factor	[71]
Immune escape	miR-325-3p	↑	LNX1	Mice/RAW264.7	Inhibiting the process of apoptosis Promoting the intracellular survival of bacteria	[77]
	miR-466	↑	TIRAP	Human/MSCs Mice/RAW264.7	Promoting macrophage polarization toward Type 2 phenotype	[78]

“↑” increased; “↓” decreased

### Regulating the invasion of the pathogen

The ability of the pathogen to induce the development of disease is closely linked with its invasion and the immune status of the host [57]. The ability of the pathogen to invade the host is considered to be the primary factor [58]. The invasive ability of the pathogen has been shown to be fine-tuned by specific miRNAs.

The adhesive potential between bacterial and alveolar epithelial cells (AECs) is enhanced by miR-155 and miR-23a [59]. A previous study indicated that the expression levels of both miR-155 and miR-23a were significantly downregulated in AECs following *Klebsiella pneumoniae* (*K. pneumoniae*) infection. In order to confirm this phenomenon, miR-155 or miR-23a mimics or inhibitors were transfected into A549 cells. The adhesive ability was evaluated by colony counting. Subsequently, it was shown that the miR-155 or miR-23a mimics effectively increased bacterial adhesion, while the application of their corresponding inhibitors reversed these results. The data implied that miR-155 and miR-23a enhance the invasion of the pathogen by strengthening its adhesion at the weakened autonomic immunity conditions.

The invasion and colonization of the bacteria were affected by miR-223-3p through regulation of the expression levels of the target CagA [60]. A previous study indicated that the expression levels of miR-223-3p in gastric cancer cells infected with *Helicobacter pylori* (*H. pylori*) were significantly higher than those without *H. pylori*. Furthermore, transfection of CagA expression vector into the cells increased the expression of miR-223-3p. The application of miR-223-3p inhibitors partially reversed CagA-mediated induction of cell proliferation and migration. Therefore, it was shown that miR-223-3p was a key mediator of *H. pylori* invasion. A recent study has also confirmed that the concentration of *H. pylori* was regulated by miR-223-3p via controlling the expression of the inflammasome nod-like receptor protein 3 (NLRP3) [61, 62].

### Modulation of the virulence of the pathogen

The virulence factor (VF) is the relative capacity of the pathogen to cause damage in host cells [63]. It is characterized by the ability of the pathogen to escape the defense mechanisms and colonize the host niche, which can cause infections in the host [64, 65]. The pathogen can survive in host cells by increasing the production of VFs [66]. The virulence of the pathogen is regulated by specific miRNAs during the process of infection. Therefore, the exploration of the role of miRNAs in the pathogenesis of VFs can provide novel options for the prevention and treatment of bacterial pneumonia.

The function of the virulence protein endopeptidase O (PepO) was adjusted by miR-155 through activation of the TLR2/NF-κB pathway [67]. PepO is a ubiquitously expressed pneumococcal virulence protein which can regulate the adhesion and invasion of *Streptococcus pneumoniae* (*S. pneumoniae*) [68]. PepO-induced phagocytosis was attenuated in cells transfected with miR-155 inhibitors, while it was ameliorated following treatment with mimics. The upregulation of miR-155 expression, which led to the induction by PepO, was mediated by TLR2/NF-κB signaling. These results indicated that miR-155 is involved in regulating the function of PepO, which could affect the virulence of the pathogen.

miR-147-3p expression is modulated by the ESAT-6 secretion system-1 (ESX-1) to mediate the secretion of VFs [69]. A previous study has shown that the secretion of VFs was mediated by ESX-1, which can aid *M. tuberculosis* to penetrate the phagosome membrane and translocate into the cytoplasm [70]. miR-147a-3p expression was downregulated during *M. tuberculosis* infection in macrophages in an ESX-1-dependent manner. To verify this phenomenon, the macrophages were transfected with miR-147 mimics or inhibitors. Consequently, the production of the inflammatory cytokines was inhibited, while the intracellular survival of *M. tuberculosis* was reduced by miR-147 mimics, and reversed by miR-147 inhibitors. In addition, the miR-147 expression levels in the ESX-1 knockout strain were upregulated. This effect was significantly higher than that noted in the non-knockout strain. The data indicated that the presence of ESX-1 was a negative regulator of miR-147. Collectively, the upregulation of miR-147-3p caused inhibition of the secretion of VFs and reduced intracellular survival.

In addition, the production of inflammatory cytokines stimulated by Rv2346c was mediated via the p38/miRNA/NF-κB axis [71]. *Mycobacterium tuberculosis* Rv2346c is a crucial VF in *M. tuberculosis* infection, which can inhibit the host immune response. A previous study demonstrated that the survival of *M. tuberculosis* was improved by Rv2346c in macrophages, which led to an increase in bacterial load and lung injury. miR-155 and miR-99b were shown to regulate the secretion of Rv2346c in *M. tuberculosis* infection by inhibiting the expression of NF-κB. Therefore, miR-155 and miR-99b may be used as targets to provide novel strategies for antibacterial therapy.

### Aiding the immune escape of the pathogen

The pathogens can survive in the host cells by evading the immune surveillance of the host via specific immune evasion mechanisms [72]. The reduction of the immune response of the host cells is considered one of the mechanisms for pathogen immune escape [73]. The classical immune response of the host includes the opsonization of phagocytes [74, 75]. Furthermore, the death of the apoptotic cells is one of the most important ways for macrophages to phagocytize the pathogens [76]. Therefore, the inhibition of host cell apoptosis can weaken the phagocytosis of macrophages, which accelerates the infection of the pathogens in the host cells. Recent studies have shown that miRNAs are involved in the regulation of apoptosis.

The immune escape of the pathogen is facilitated by the expression of miR-325-3p, which promotes the anti-apoptotic STAT3 signaling [77]. Earlier studies have shown that miR-325-3p expression is significantly upregulated following infection of *M. tuberculosis*. Subsequent studies confirmed that the ligand of numb protein-X1 (LNX1), an E3 ubiquitin ligase of NIMA-related kinase 6 (NEK6), is a direct target of miR-325-3p. The proteasomal degradation of NEK6 can be hampered by LNX1 in macrophages. The anti-apoptotic signaling of STAT3 is activated via the abnormal accumulation of NEK6, which can inhibit the process of apoptosis, promote the immune escape of *M. tuberculosis*, and increase its intracellular survival. To verify these associations, miR-325-3p mimics or inhibitors were transfected into macrophages. It was shown that the proliferation and intracellular survival of *M. tuberculosis* was increased following the transfection of miR-325-3p mimics into the cells, where it was inhibited following their treatment with inhibitors. Concomitantly, these effects have also been confirmed in mice transfected with miR-325-3p mimics. Taken together, the data indicated that miR-325-3p contributes to the immune escape of the pathogen, which provides a theoretical basis for the development of therapeutic approaches for bacterial resistance.

By regulating the immune microenvironment in the host–pathogen interaction, the miRNAs are involved in regulating the entire process of bacterial drug resistance. miR-466 has demonstrated certain therapeutic effects in pneumonia patients infected with the multidrug-resistant *P. aeruginosa* (MDR-PA) strain [78]. It has also been shown that miR-466 is highly expressed in mesenchymal stromal cells (MSCs) and MSC-derived extracellular vesicles (MSC EVs). miR-466 mimics were transfected into macrophages. It was shown that the expression levels of mRNAs coding for M2-related arginase-1 (Arg-1) and interleukin 10 (IL-10) were substantially increased, whereas those coding for M1-related inducible nitric oxide synthase (iNOS) and IL-12 were significantly decreased. In addition, the number of intracellular bacteria was increased. The vital role of miR-466 in the regulation of the balance of the M1/M2 macrophage phenotype was established. In addition, the expression levels of the Toll-interleukin 1 receptor domain containing adaptor protein (TIRAP) were inhibited by miR-466. In macrophages transfected with miR-466 mimics, the expression levels of both TIRAP and the protein myeloid differentiation primary response protein 88 (MyD88) were reduced, whereas the expression levels of the key proteins involved in the NF-κB pathway were augmented. Overall, both MSCs and MSC EVs regulated by miR-466 exhibited certain therapeutic effects on MDR-PA-induced pneumonia. These results demonstrate a potential novel therapeutic area for the treatment of multidrug-resistant hospital-acquired pneumonia (HAP) and ventilator-associated pneumonia (VAP).

### Application in bacterial pneumonia

**Fig. 4:**
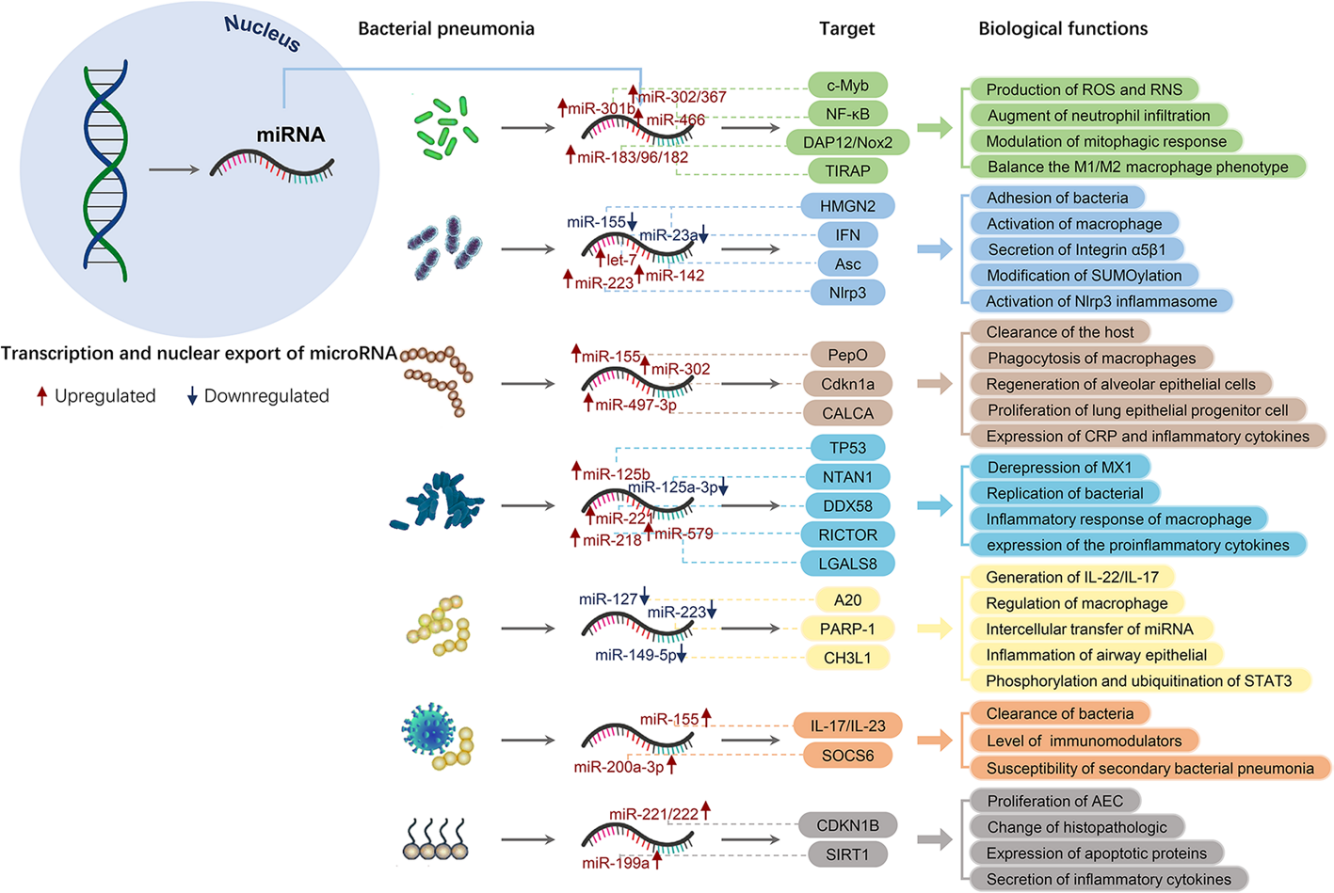
Examples of miRNAs and their targets and biological functions in bacterial pneumonia

The abnormal expression of miRNAs plays an important role in the development of specific diseases, notably in infectious diseases and has been the major focus of investigation of previous research studies [79]. It was shown that the expression levels of certain miRNAs varied with the progression of bacterial pneumonia (Fig. 4). The miRNA methodological techniques of reverse inhibition of expression have been widely used in related research studies.

### *P. aeruginosa*-induced pneumonia

*Pseudomonas aeruginosa* is an opportunistic pathogen. It is not only the main cause of disability and death in hospitalized patients with hypoimmunity [80], but also the major reason of chronic airway infection in patients with CF [81], bronchiectasis [82] and chronic bronchitis [83]. Antibiotics are the main drugs used to treat bacterial infections. However, due to the intrinsic and available resistance mechanisms of *P. aeruginosa*, the efficacy of antibiotic treatment is limited [84]. Recently, certain miRNA techniques have been used to confirm that miRNAs are involved in the immune regulation and anti-infection reaction of the host. This evidence can be used to offer new prospects for the diagnosis and treatment of *P. aeruginosa*-induced pneumonia.

The inflammation of *P. aeruginosa* infection was augmented by miR-301b via inhibition of the expression of c-Myb [85]. A previous study indicated that the expression levels of miR-301b were induced with *P. aeruginosa* infection via the TLR4/MyD88/NF-κB pathway. Transfection of the miR-301b mimics and inhibitors into *P. aeruginosa*-infected macrophages resulted in the identification of c-Myb as the target of miR-301b. Moreover, the number of anti-inflammatory cytokines was positively regulated by c-Myb, while the number of proinflammatory cytokines exhibited a negative regulation. The excessive inflammation and impaired host defense were mediated by miR-301b mimics via the decrease of the transcription of c-Myb, while it was reversed by miR-301b inhibitors. It was also confirmed that the increased neutrophil infiltration was caused by the repression of miR-301b in vivo, which alleviated the infectious symptoms in mice. Therefore, a novel mechanism for balancing inflammation was provided in response to bacterial infection.

The function of macrophages was modulated by the miR183/96/182 cluster to *P. aeruginosa* [86]. It was found that the expression levels of miR-183/96/182 in macrophages from *P. aeruginosa* were increased. The miRNA replacement therapy can reduce the production of ROS and reactive nitrogen species (RNS) in macrophages infected with *P. aeruginosa*. Specifically, this reduction was noted following transfection of the cells with miR-183/96/182 mimics. However, the production of proinflammatory cytokines was decreased following knockdown of the expression of the cluster. In addition, it was also shown that the DNAX activation protein of 12 kDa (DAP12) and the NADPH oxidase 2 (NOX2) were the downstream target genes of the miR-183/96/182 cluster. Knockdown or inhibition of the expression of miR-183/96/182 in macrophages can contribute to the enhancement of the bactericidal capacity. Therefore, the miR183/96/182 cluster exerted a significant potential to combat antibiotic-resistant bacterial infection by strengthening innate immunity.

The host anti-microbial defense was investigated by the miR-302/367 cluster via the modulation of the mitophagic response against *P. aeruginosa* infection [87]. The infection of alveolar macrophages by *P. aeruginosa* caused a significant increase in the expression of the miR-302/367 cluster. The mitophagic response was accelerated by miR-302/367 mimics, which increased the clearance of *P. aeruginosa* by regulating the production of ROS. However, miR-302/367 inhibitors exhibited the opposite effects. Subsequent studies confirmed that the induction of mitophagy and the bacterial clearance were associated with the induction of NF-κB, a negative regulator of mitophagy. The process of mitophagy was accelerated by inhibiting NF-κB expression. Moreover, inhibition of NF-κB attenuated the production of ROS or cytokines, which could reduce cell injury by *P. aeruginosa* infection. Collectively, the results demonstrated that the miR-302/367 cluster played an effective regulatory role in the anti-microbial defense of *P. aeruginosa* mediated by mitophagy. The miR-302/367 cluster may be used as a potential target for the development of treatment and intervention for *P. aeruginosa*-related diseases.

As previously mentioned in the section of the immune escape of the pathogen, miR-466 exhibited certain therapeutic effects in pneumonia from MDR-PA by regulating the immune microenvironment in the host–pathogen interaction [78].

miR-636 is a novel actor for the regulation of the pulmonary inflammation in CF [88]. It was found that miR-636 was overexpressed in CF patients. miR-636 was directly related to the expression of proinflammatory cytokine receptors (IL1R1, RANK) and of the major protein in the NF-κB pathway (IKBKB). Specifically, the miRNA inhibition and replacement therapy caused an increase in the expression levels of the RANK protein, whereas the activity levels of the NF-κB and the secretion of IL-6/IL-8 were decreased following transfection of miR-636 mimics into CF cells. However, opposite effects were noted with the antagomiR-636. Therefore, it was confirmed that miR-636 was involved in the regulation of the inflammatory response of CF. However, a subsequent study demonstrated that *P. aeruginosa*, the main colonizer of CF patients, was not bound with miR-636, which indicated that not all related miRNAs were regulated by bacteria. In brief, the data indicated that miRNA regulation is a huge and complex network.

### *K. pneumoniae*-induced pneumonia

*K. pneumoniae* is the main pathogen found in hospitals and community-acquired infections [89]. It is more challenging to select appropriate antibiotic treatment with the rapid acquisition of antibiotic resistance [90, 91]. Carbapenem resistance was the biggest threat to *K. pneumoniae* treatment [90]. Previous studies have predicted that if 15.2% of *K. pneumoniae* were resistant to carbapenems, the mortality of untreated *K. pneumoniae* infections in the hospital could be as high as 50% [90]. Based on this evidence, an urgent need is required for the development of novel methods for the treatment of *K. pneumoniae*-induced pneumonia.

The adhesion of *K. pneumoniae* was modulated by miR-155 and miR-23a via integrin-mediated α5β1 signaling [59]. An earlier study demonstrated that the expression levels of miR-155 and miR-23a were downregulated in pulmonary epithelial cells with *K. pneumoniae*. The function of integrin α5β1 was promoted by miR-155, which led to the increase of actin polymerization. Moreover, the expression levels of high mobility group nucleosomal-binding domain 2 (HMGN2), which is the potential target of miR-155 and miR-23a, were involved in regulating the α5β1 expression and *K. pneumoniae* adhesion. It was confirmed that HMGN2 expression was significantly inhibited by miR-155 and miR-23a mimics, while the opposite results were noted by miR-155 inhibitors. In brief, the adhesion of *K. pneumoniae* was regulated by miR-155 and miR-23a by targeting HMGN2 to mediate the activation of integrin α5β1. These results revealed a novel connection between miRNAs and integrin function that can regulate bacterial adhesion.

The NLRP3 inflammasome activation is suppressed by the miR-223/142 axis via inhibition of NLRP3 and ASC [92]. To elucidate the function of microvesicle (MV)-containing miR-223/142 in lung inflammation, miR-223/142 mimics were transfected into unstimulated MVs, which were delivered into the murine lungs. It was found that the lung macrophages were selectively affected by the miR-223/142 mimics. The inflammatory lung responses triggered by *K. pneumoniae* were suppressed by overexpression of miR-223/142. A further study indicated that the activation of the NLRP3 inflammasome in macrophages was synergistically suppressed by miR-223 and miR-142 via the inhibition of NLRP3 and ASC. Therefore, the activation of macrophages and the induction of lung inflammation were attenuated by miR-223/142 via vesicle-mediated delivery. Collectively, the results indicated that miR-223/142 may be a novel diagnostic marker for pulmonary inflammation induced by Gram-negative bacteria.

Let-7 miRNAs are involved in the regulation of SUMOylation to modulate the activation of the immune defense [93]. The SUMOylation of host proteins in epithelial cells and macrophages was decreased following *K. pneumoniae* infection. It was found that the increase in SUMOylation levels was caused by the inhibition of let-7f and let-7 g in the cells with *K. pneumoniae*. The inhibition of let-7f and let-7 g expressions significantly reduced the number of bacteria present. Therefore, an increase in SUMOylation levels can inhibit infection by promoting the activation of the inflammatory response and decreasing the intracellular survival of the macrophages. Collectively, the results of that study demonstrated a strategy employed by *K. pneumoniae* to modulate the activation of immune defense by controlling the modification of the proteins.

### *S. pneumoniae*-induced pneumonia

*Streptococcus pneumoniae* is a common Gram-positive bacterium, which colonizes the upper respiratory tract [90]. The high incidence of *S. pneumoniae* [94], the shortcomings of capsular serotype vaccine [95] and the increasing resistance of antibiotics [96] have resulted in the increased requirement to identify and develop novel and effective prevention and treatment methods for this pathogen. Therefore, the assessment of the exact mechanism of immune transduction is imperative for providing effective treatment against *S. pneumoniae*-induced pneumonia.

The phagocytosis of the macrophages can be enhanced by PepO in a miR-155- and TLR2-dependent manner [67]. *Streptococcus pneumoniae* PepO is a newly discovered and widely expressed pneumococcal virulence protein, which can enhance the phagocytosis of *S. pneumoniae* through the peritoneal exudate macrophage (PEMs). A previous study demonstrated that miR-155 expression was upregulated in PepO-induced PEMs. Moreover, it was confirmed that PepO-induced phagocytosis of PEMs was attenuated in cells transfected with miR-155 inhibitors, whereas this effect was ameliorated with miR-155 mimics. Furthermore, it was verified that the upregulation of miR-155 expression was mediated via the TLR2/NF-κB pathway. Therefore, the clearance of the host could be enhanced by miR-155 in PepO-induced PEMs.

The host recovery from pneumonia caused by *S. pneumoniae* was enhanced by miRNA-302 via induction of AEC regeneration [97]. A previous study indicated that the substantial damage to AECs in *S. pneumoniae*-infected mice was a slow process of regeneration. In addition, the expression levels of miRNA-302 in AECs were increased, while AECs were regenerated. In order to clarify the exact mechanism of action, miR-302 mimic therapy was used to treat *S. pneumoniae*-infected mice. The proliferation of lung progenitor cells and the regeneration of AECs were successfully accelerated by this therapy. The data indicated that miRNA mimic therapy could be used to enhance the host’s recovery from bacterial pneumonia. Therefore, this can be used as a novel application of regenerative medicine for the treatment of microbial infection.

The inflammatory response of bacterial pneumonia can be mediated by miR-497-3p via the regulation of the expression of procalcitonin (PCT) [98]. Earlier findings indicated that the expression levels of miR-497-3p and PCT were increased in the established bacterial pneumonia mouse model and in the clinical samples. The PCT levels were positively correlated with miR-497-3p expression. Moreover, calcitonin (CALCA), which is the gene that encodes PCT, was identified as the functional target of miR-497-3p. In mice infected with *S. pneumoniae*-induced pneumonia, the inhibition of miR-497-3p significantly attenuated the expression levels of C-reactive protein (CRP) and inflammatory cytokines in the lung tissues. Collectively, the data indicated that miR-497-3p may be considered as a potential target for the treatment of bacterial pneumonia in the clinic.

### *L. pneumophila*-induced pneumonia

*Legionella pneumophila* (*L. pneumophila*) is a Gram-negative bacterium, which mainly exists in freshwater environments [99]. The main route of *L. pneumophila* infection in humans is via the inhalation of contaminated aerosols [100]. Once inhaled into the lungs, severe pneumonia can be caused by *L. pneumophila* infection through resident alveolar macrophages [101]. *Legionella pneumophila*-induced pneumonia is easily misdiagnosed due to the insignificant differences in the symptoms of the respiratory tract infection, which suggests that these patients cannot be treated timely and effectively [102]. The application of miRNA-based methods might help to clarify the pathogenesis of *L. pneumophila*, and might provide one of the specific markers for the detection of this bacterium.

The inflammatory response of macrophages against *L. pneumophila* infection is mediated by miR-218 [103]. During *L. pneumophila* infection, the expression levels of miR-218 were upregulated and the expression levels of rapamycin-insensitive companion of mTOR (RICTOR) were downregulated. In order to elucidate the relationship between miR-218 and RICTOR, miR-218 mimics were transfected into U937 cells. As a result, the expression levels of the RICTOR protein were inhibited by the overexpression of miR-218, while knockdown of miR-218 expression by specific inhibitors restored this effect. Furthermore, the expression levels of the proinflammatory cytokines IL-6 and tumor necrosis factor alpha (TNF-α) were induced by *L. pneumophila* infection. This effect was regulated by the knockdown of the expressions of miR-218 or RICTOR. Collectively, the findings indicated that miR-218 and RICTOR exhibit potential roles as therapeutic targets for *L. pneumophila* infection.

A previous study indicated that the replication of *L. pneumophila* was regulated by miR-125a-3p through targeting of NTAN1 [104]. An additional study indicated that the replication of *L. pneumophila* was also controlled by the miR-125b/221/579 network via galectin-8 (LGALS8) and MX dynamin-like GTPase 1 (MX1) [105]. Specifically, the upregulation of miR-125b/221/579 expression was induced by *L. pneumophila* infection. The miRNA inhibition and replacement therapy demonstrated that miR-221, miR-125b, and miR-579 targeted DExD/H-box helicase 58 (DDX58), tumor protein P53 (TP53), and LGALS8, respectively. Moreover, the transfection with the miRNA inhibitors resulted in reduced intracellular replication, while treatment with all three miRNA precursors resulted in a strong increase of intracellular replication. MX1 was also involved in the miRNA-dependent control of *L. pneumophila* replication. Therefore, the present study clarified a cell-autonomous immune network to restrict the replication of *L. pneumophila*.

### *S. aureus*-induced pneumonia

*Staphylococcus aureus* is one of the most common nosocomial pathogens [106]. The incidence of methicillin-resistant *S. aureus* (MRSA) accounts for 20–40% of HAP and VAP and has increased rapidly during the last few years [107]. Generally, secondary *S. aureus* infections such as organ abscesses and sepsis are harder to cure than skin and soft tissue infections [108, 109]. In addition, the increasing antibiotic resistance has made the treatment of *S. aureus* pneumonia more difficult and expensive [110]. Therefore, novel alternatives to prevent and treat *S. aureus* infections are desperately needed. Recently, various miRNAs have been identified that play an essential role in infection by *S. aureus*.

The anti-microbial host defense is mediated by miR-127 via targeting the A20/STAT3 axis [55]. By using miR-127 mimics and inhibitors, it was confirmed that miR-127 conferred protection against *S. aureus* pneumonia. The regulation of macrophages and the production of IL-22/IL-17 are the main reasons for its protective effect. In addition, STAT3 can initiate the transcription of IL-22/IL-17 in response to infectious or inflammatory signals. Therefore, the impaired expression of IL-17/IL-22 could be led by STAT3 loss-of-function, which renders the host more susceptible to bacterial infection. It is important to note that A20, a target of miR-127, induces downregulation of the phosphorylation and ubiquitination of STAT3, leading to compromised antibacterial responses. Therefore, miR-127 is the key modulator of the host defense against *S. aureus* infection.

Acute lung injury (ALI) can be dampened by the intercellular transfer of miR-223 [111]. Previous studies demonstrated that the cross-talk of neutrophils and lung epithelial cells could dampen inflammation. miR-223 can be transferred from neutrophils to lung epithelial cells. To assess the relationship between miR-223 and ALI, the miR-223 mimics were injected into the trachea of wild-type mice. It was found that the pulmonary edema and lung inflammation were attenuated by the overexpression of miR-223. In addition, PARP-1, which is a miR-223 target gene, could facilitate inflammatory responses by activating specific transcription factors. Therefore, these studies indicated that miR-223 exhibits protective effects in the acute phases of lung injury and infection.

Recently, certain miRNA-based methods were used to prove that miR-149-5p was involved in regulating NF-κB-mediated inflammation of airway epithelial cells by targeting chitinase 3-like 1 (CH3L1) [112].

### Co-infection of viral and bacterial pneumonia

Viral and bacterial co-infections are common in community-acquired pneumonia [113]. Generally, severe pneumonia may be due to the virus itself. However, death from pneumonia is usually related to secondary bacterial infections [114]. For example, nearly 30% of 2009 influenza A (H1N1) pandemic cases in the United States were related to secondary bacterial infections [115]. Approximately 55% of these were fatal cases [116]. Therefore, secondary bacterial infections of viral pneumonia were the main reasons for the aggravation of pneumonia [117, 118]. miRNAs have been shown to play vital roles in the pathogenesis and treatment of viral and bacterial co-infection.

The host immune response of co-infection pneumonia was found to be regulated by miR-155 via the IL-23/IL-17 pathway [119]. To clarify the associated mechanism of action, mice were sequentially infected with influenza and MRSA. It was found that miR-155 levels were increased, whereas those of IL-17/IL-23 were decreased following co-infection. Subsequently, miR-155 antagomir was used to treat co-infected mice. The data indicated that the clearance of bacteria in the lungs was increased 4.2-fold. In addition, this trend of changes was consistent with the results collected from patients with post-viral bacterial pneumonia. Collectively, the results indicated that the post-influenza immune response to subsequent bacterial challenge was modulated by miR-155 through the IL-17/IL-23 pathway in the lung. Therefore, the antagonism of certain miRNAs may be a potential therapeutic strategy to combat secondary bacterial pneumonia.

The expression of chemokine (C-X-C motif) ligand 10 (CXCL10) was shown to be indirectly modulated by miR-200a-3p by targeting suppressor of cytokine signaling-6 (SOCS-6) to regulate the immune response to co-infection [120]. A previous study indicated that the expression of miR-200a-3p was induced following co-infection with influenza virus and *S. pneumoniae*. Moreover, the application of the miRNA inhibition and replacement therapy has shown that miR-200a-3p expression is positively associated with CXCL10 expression and negatively associated with SOCS-6. In addition, the expression levels of SOCS-6, which is a regulator of the JAK-STAT pathway and of CXCL10, which is an interferon-γ-induced protein, were associated with viral and bacterial co-infection and pneumonia severity. Therefore, the monitoring and evaluation of serum SOCS-6 and CXCL10 levels induced by miR-200a-3p can aid the diagnosis and treatment of severe co-infection pneumonia.

### Pneumonia due to LPS

Lipopolysaccharide (LPS), which is also called endotoxin, exhibits a unique structure in the cell wall of Gram-negative bacteria [121]. It is the main pathogenic substance caused by bacterial infection [122]. LPS can be released during the lysis of bacterial cells, which may induce a series of nonspecific inflammatory cytokines [123]. Currently, LPS is known as an inducer of oxidative damage and has been widely used to establish animal or cell models of inflammation and bacterial infection [124, 125]. Therefore, the role of miRNAs in LPS-induced pneumonia has a certain degree of universal applicability.

The proliferation of lung epithelial cells was promoted by miR-221/222 via targeting of the cyclin-dependent kinase inhibitor 1 B (CDKN1B) pathways [126]. A previous study indicated that LPS, a stimulator of bacteria, caused significant upregulation in the expression levels of miR-221/222 in macrophage-derived apoptotic bodies (ABs). In addition, the proliferation of lung epithelial cells was promoted by macrophage-derived ABs. To confirm the association between miR-221/222 and lung epithelial cells, miR-221 and/or miR-222 inhibitors were transfected into the LPS-induced ABs. It was found that the AB-mediated proliferation of lung epithelial cells was significantly reduced with the deletion of miR-221/222. Similarly, miR-221/222 mimics or inhibitors were introduced into LPS-induced macrophage-derived ABs, which confirmed that the expression levels of CDKN1B were inhibited by miR-221/222. Therefore, AB-shuttling of miR-221/222 could promote cell growth by regulating the CDKN1B pathways. Collectively, the data indicated that the proliferation of lung epithelial cells is promoted by AB-shuttling of miRNA-221/222 via targeting of CDKN1B. Therefore, miR-221/222 is expected to be the key to innate immune protection against bacterial pneumonia.

Sepsis-induced ALI was attenuated by miR-199a via targeting of sirtuin 1 (SIRT1) [127]. It was found that miR-199a expression could be upregulated by LPS in alveolar macrophages. The upregulation of miR-199a expression stimulated the secretion of inflammatory cytokines and enhanced the expression of the apoptotic proteins. Therefore, the inhibition of miR-199a indicated a protective role for ALI. In addition, miRNA inhibition and replacement therapy confirmed that SIRT1, which is a direct target of miR-199a, was negatively associated with miR-199a expression. The protective role of the downregulation of miR-199a expression was attenuated by treatment with SIRT1 inhibitors. Collectively, the data indicated that miR-199a exhibited potential therapeutic effects in sepsis-induced ALI, which provides a theoretical basis for the development of new miR-199a-targeting drugs against ALI.

### Challenges and limitations

miRNAs can be used as technical tools for gene function research [128]. Compared with siRNA, miRNAs are highly conserved, time-ordered, and tissue-specific [129]. It is a mechanism by which organisms regulate the post-transcriptional expression of target genes [130]. Although well-designed miRNAs can provide long-term silencing, they exhibit reduced side effects and are safer to use compared with siRNAs [14]. In recent years, miRNA techniques have demonstrated enormous development and utilization value. However, the current application of miRNA techniques faces several challenges, such as low sensitivity, poor specificity, low silencing efficiency, off-target effects, and toxic reactions [131, 132]. In addition, the majority of the applications of the miRNA techniques and the research of miRNAs are at an early stage of development [133]. Therefore, this method requires further improvement in future studies.

*Low sensitivity* The short length of mature miRNA sequences, the high similarity of homologous sequences, and the low abundance and high susceptibility to degradation have led to the challenging of the high sensitivity of the miRNA-based methods [134]. The latest research demonstrated that the amplification of miRNA biosensors using DNA nanotechnology could effectively amplify the recognition signal and improve the sensitivity of miRNAs [135]. This suggests that DNA nanotechnology exhibits a high potential to enhance the sensitivity of the miRNA-based methods [136]. It has been reported that an amplification-free multi-color single-molecule imaging technique can discriminate single base mismatches and single-nucleotide 3′-tailing with low false positive rates on miRNA [137]. This technique can profile purified endogenous miRNAs with high sensitivity. Besides, combined CRISPR-based approaches for miRNA detection with the benefits of enzyme-assisted signal amplification and enzyme-free amplification biosensing technologies could provide high sensitivity in diagnosis [138].

*Poor specificity* Generally, miRNAs expressed in one or two certain tissues are considered to be tissue-specific [136]. However, previous studies have shown that the majority of the miRNAs are not expressed in one tissue, but in multiple tissues and/or organs. Furthermore, no standard consensus has been reported for the identification of tissue-specific miRNAs [136, 139, 140]. The limitations of the poor specificity impeded the delivery of miRNA mimics into target cells. The limitations include poor stability in vivo, irrational bio-distribution, endogenous RNA disruption, and potential side effects [141]. These limitations also lead to the poor specificity of miRNAs with regard to the targeting of specific tissues and organs and subsequently limit their clinical application [142].

*Low silencing efficiency* The silencing efficiency of miRNAs is lower than that of siRNA [143]. siRNAs are complementary to the target mRNA. Specifically, miRNAs combine with target RNAs, which contain a single-stranded nucleic acid form. This combination is not completely complementary and is prone to mismatch [9, 144]. The methods for assessing the mechanism of miRISC inhibition of target mRNA translation have been well characterized. However, the specific mechanism of inhibition remains controversial. Therefore, improvement of the efficiency of miRNA silencing is still a major focus of investigation.

*Off-target effect* Since the length of the seed sequence is only 6–8 nt, the overexpression of miRNAs usually contains multiple targets. Therefore, it is difficult to predict the sites of nonspecific targets [145]. Off-target effects are possible to occur, understanding off-target effects as the key to successful RNAi therapy [146]. Fortunately, it was reported that the interaction between genome-wide association and AGO-RIP seq could effectively control the miRNA off-target effects [147]. In addition, the miRNA functions can be effectively inhibited by the circular antisense microRNA oligodeoxynucleotides (AMOs) with low off-target effects [148]. Binding and enzymatic assays indicated that the topological properties of circular c-ODNs significantly decreased their off-target effects as miRNA inhibitors [148].

*Toxic reaction* The cytotoxicity of transfection reagents, including miRNA agonists and antagonists, is responsible for the adverse side effects of the miRNA-based methods [149]. Excessive toxicity can directly affect the survival, proliferation, migration, and apoptosis of experimental cells, and seriously influence experimental results. In addition, the off-target inhibition from off-target effects can cause unwanted toxicity. These factors increase the uncertainty of using miRNAs in research and diagnostic and therapeutic applications, and can cause uncontrollable harm to model organisms [147].

*Experimental use of miRNAs* At present, the miRNA-based methods used in bacterial pneumonia are still in their infancy. A limited number of these methods can be used in clinical practice [133, 150]. Although the development of miRNA-based methods faces unparalleled challenges, many strategies for clinical practice are being explored, like RNA targets for small molecules and RNA drugs. It has been reported that many natural, semisynthetic, or synthetic antibiotics (e.g., tetracyclines, aminoglycosides, oxazolidinones, macrolides, and phenicols) can directly combine with ribosomal RNAs to achieve the inhibition of bacterial infections [151].

Collectively, the data indicated that although miRNA-based methods have seen considerable advancements, the aforementioned obstacles have to be overcome. Despite these limitations, they are considered to be promising diagnostic and therapeutic tools.

## Conclusion

miRNAs play an important role in the complex interaction between host and pathogen due to their ability to regulate gene expression at the post-transcriptional level. miRNAs act as part of the host immune response to neutralize pathogen infection [39]. Moreover, they can act as bacterial accomplices to induce pathogen infection [39]. Recently, several experimental studies have shown that specific miRNAs are involved in regulating the interaction between host and pathogen via inhibition and/or replacement therapy, including the recognition, defense, and clearance of the host, as well as the invasion, virulence, and immune escape of the pathogen. It was shown that miRNAs were involved in regulating the recognition of PAMPs by TLRs to activate the initial modulation of the host’s resistance to pathogen invasion. miRNAs are involved in the regulation of host innate and adaptive immunity and act as host defense monitoring factors. They also adjust the polarization of macrophages to regulate the host’s ability to eliminate bacteria. miRNAs modulate the invasion of bacteria by fine-tuning the pathogen-induced immune response and self-defense mechanism. They also modulate the virulence of bacteria by regulating the production of VFs. Finally, they can aid the immune escape of bacteria by regulating the apoptosis of host cells.

In addition, miRNA-based methods are widely used in the research of bacterial pneumonia. miRNA techniques have confirmed that a variety of miRNAs are key targets for the diagnosis, treatment, and prognosis of pneumonia via inhibition and/or replacement therapy. For example, the proinflammatory response of bacterial infection was augmented by miR-301b in *P. aeruginosa*-induced pneumonia; the phagocytosis of macrophages was enhanced by miR-155 in *S. pneumoniae*-induced pneumonia; bacterial adhesion was modulated by miR-155 and miR-23a in *K. pneumoniae*-induced pneumonia; bacterial replication was regulated by miR-125a-3p in *L. pneumophila*-induced pneumonia. Furthermore, miR-302 in *S. pneumoniae*-induced pneumonia and miR-221/222 in LPS-induced pneumonia could promote the proliferation of AECs. The expression levels of miR-29b-2-5p were significantly different in the early and late stages of bacterial infection. These two miRNAs played a dual regulatory role in the prevention of bacterial infection. The aforementioned miRNAs can be used as key targets for the progression and prognosis of bacterial pneumonia. Collectively, the results have indicated that the use of miRNA techniques to explore novel methods for the diagnosis and treatment of bacterial pneumonia is an effective way to treat severe cases of bacterial pneumonia.

Bacterial pneumonia is a serious threat to human health. Due to its unavoidable resistance to antibiotics, the discovery and application of diagnostic and prognostic markers will greatly reduce the mortality of "refractory" or drug-resistant bacterial pneumonia. In the past few decades, the role of miRNAs in the pathogenesis, diagnosis, and treatment of bacterial pneumonia has been gradually clarified. Recently, the extensive and comprehensive utilization of miRNA-based methods has provided additional comprehensive theoretical support in the study of the pathogenesis of bacterial pneumonia. This in turn offers more opportunities for the treatment of bacterial pneumonia. However, these studies were mainly supported by experimental laboratory findings. A limited number of miRNA molecules have been assessed in clinical trials, and almost none of them demonstrated significant progress. Although miRNAs have been used in the study of bacterial pneumonia infection and pathogenesis, their sensitivity and specificity seem to be unsatisfactory. Therefore, in the future, a series of challenges must be resolved in the clinical application of miRNA-based methods for the treatment of bacterial pneumonia. These include off-target effects related to hybridization, difficulties in transmembrane delivery of miRNA in vivo, and sequence-independent toxicity from nucleic acid drugs. The resolution of these challenges will be the key to the universal application of miRNA-based methods in the treatment of pneumonia in the future.

## Data Availability

Data sharing is not applicable to this article as no datasets were generated or analysed during the current study.

## Abbreviations

miRNAs: MicroRNAs
RNAi: RNA interference
dsRNA: Double-stranded RNA
siRNAs: Small interfering RNAs
mRNA: Messenger RNA
3′-UTR: 3′-Untranslated region
pri-miRNAs: Primary miRNAs
Pol II&III: Polymerase II and III
pre-miRNAs: Precursor miRNAs
RISC: RNA-induced silencing complex
miRISC: MiRNA-induced silencing complex
PAMPs: Pathogen-associated molecular patterns
PRRs: Pattern-recognition receptors
TLRs: Toll-like receptors
TLR4: Toll-like receptor 4
*M. tuberculosis*: *Mycobacterium tuberculosis*
CD: Crohn’s disease
CF: Cystic fibrosis
*P. aeruginosa*: *Pseudomonas aeruginosa*
ROS: Reactive oxygen species
*S. aureus*: *Staphylococcus aureus*
AECs: Alveolar epithelial cells
*K. pneumoniae*: *Klebsiella pneumoniae*
*H. pylori*: *Helicobacter pylori*
NLRP3: Nod-like receptor protein 3
VF: Virulence factor
PepO: Protein endopeptidase O
*S. pneumoniae*: *Streptococcus pneumoniae*
ESX-1: ESAT-6 secretion system-1
LNX1: Ligand of numb protein-X1
MDR-PA: Multidrug-resistant *P. aeruginosa*
MSCs: Mesenchymal stromal cells
MSC EVs: MSC-derived extracellular vesicles
Arg-1: Arginase-1
IL-10: Interleukin 10
TIRAP: Toll-interleukin 1 receptor domain containing adaptor protein
MyD88: Myeloid differentiation primary response protein 88
HAP: Hospital-acquired pneumonia
VAP: Ventilator-associated pneumonia
RNS: Reactive nitrogen species
DAP12: DNAX activation protein of 12 kDa
NOX2: NADPH oxidase 2
HMGN2: High mobility group nucleosomal-binding domain 2
MV: Microvesicle
PEMs: Peritoneal exudate macrophage
PCT: Procalcitonin
CALCA: Calcitonin
CRP: C-reactive protein
*L. pneumophila*: *Legionella pneumophila*
RICTOR: Rapamycin-insensitive companion of mTOR
TNF-α: Tumor necrosis factor alpha
LGALS8: Galectin-8
MX1: MX dynamin-like GTPase 1
DDX58: DExD/H-box helicase 58
TP53: Tumor protein P53
MRSA: Methicillin-resistant *S. aureus*
ALI: Acute lung injury
CH3L1: Chitinase 3-like 1
H1N1: Influenza A
CXCL10: Chemokine C-X-C motif: ligand 10
SOCS-6: Cytokine signaling-6
LPS: Lipopolysaccharide
CDKN1B: Cyclin-dependent kinase inhibitor 1 B
ABs: Apoptotic bodies
SIRT1: Sirtuin 1
AMOs: Antisense microRNA oligodeoxynucleotides
